# “Flying a plane and building it at the same time”: Lessons learned from the dynamic implementation of mass vaccination clinics in the Region of Waterloo, Ontario, Canada

**DOI:** 10.1186/s12961-023-01036-z

**Published:** 2023-10-03

**Authors:** Moses Tetui, Ryan Tennant, Maisha Adil, Arthi Bala, Catherine Burns, Nancy Waite, Kelly Grindrod

**Affiliations:** 1 School of Pharmacy, University of Waterloo, Kitchener, Ontario Canada; 2 School of Public Health Sciences, University of Waterloo, Waterloo, Ontario Canada; 3 Department of Epidemiology and Global Health, Umeå University, Umeå, Sweden; 4 Systems Design Engineering, Faculty of Engineering, University of Waterloo, Waterloo, Ontario Canada

**Keywords:** COVID-19 pandemic, Mass vaccination clinics, Dynamic implementation, Multi-disciplinary teams, Rapid evolution, Unpredictable, Adaptable

## Abstract

**Background:**

Vaccination plays a critical role during pandemics, and mass vaccination clinics are often an imperative public health measure. These clinics usually consist of multi-disciplinary teams, which can pose significant coordination challenges, yet also present an opportunity for collectively contributing towards mitigating the impact of infection within communities. This study explores the coordination dynamics of the Region of Waterloo’s coronavirus disease of 2019 (COVID-19) mass vaccination clinics in Ontario, Canada, between July 2021 and April 2022.

**Methods:**

This qualitative study included 16 purposively selected participants working in mass vaccination clinics. Participants were individually interviewed for 40–60 min. An inductive and iterative thematic analysis was undertaken, including open coding, grouping, labelling, regrouping and making sense of the themes.

**Results:**

Three interrelated themes were created: (1) unpredictable work environment, which was comprised of changing clinic processes and the impact of clinic adjustments to the running of the clinics; (2) clinic cohesion challenges, which included staff role disparities, limited job preparation and clinic system silos; and (3) adaptable and supportive work environment, which was comprised of staff adaptability, dispositional flexibility and a supportive work environment. While the first two themes created a precarious situation in the clinics, the third countered it, leading to a largely successful clinic implementation.

**Conclusions:**

The rapid evolution and high transmissibility of COVID-19 in communities required a public health response that felt like flying and building a plane simultaneously – a seemingly impossible yet necessary task. However, an adaptable and supportive work environment was critical for establishing an atmosphere that can overcome challenges from a constantly changing pandemic and the guidance of public health officials. Such lessons gained from understanding the dynamic experiences in mass vaccination clinics are essential for improving the development and operation of future immunization campaigns.

## Background

The development of the coronavirus disease 2019 (COVID-19) vaccines has been an unprecedented and unpredictable feat. On 11 March 2020, the World Health Organization declared COVID-19 a global pandemic [1], and less than 9 months later, on 9 December 2020, Health Canada approved the first vaccine for emergency use [2]. COVID-19 immunizations have since played a significant role in reducing the impact of severe acute respiratory syndrome coronavirus 2 (SARS-CoV-2) on public health, despite its evolution, by minimizing hospitalizations [3, 4], severe illness [5–7] and deaths [4, 8]. However, going from vaccine approval to rapidly implementing mass vaccination clinics in Canada’s various provinces and regions also required an unprecedented collaboration among many stakeholders. Learning from this experience is vital to improving future mass immunization campaigns.

When vaccines are available, and there is high demand for vaccination, public health organizations face considerable pressure to implement immunization campaigns quickly. Mass vaccination clinics are one effective approach [9, 10], requiring multi-disciplinary stakeholders, including public health nurses, pharmacists, physicians, non-clinical staff, students and volunteers [11, 12]. These stakeholders ensure that the immunization environment meets the pandemic and the community’s unpredictable needs and safety requirements [10, 13], while simultaneously preparing themselves and other clinic staff for their respective roles despite potential barriers [14]. During the initial COVID-19 immunization campaign, the implementation of mass vaccination clinics has highlighted potential operational challenges, such as balancing the required positions and operations with community demand [10], staff scheduling [15], and the importance of proactive communication methods among staff to support shared situational awareness [16], all of which must be addressed to improve mass vaccination clinics as a functioning system.

Understanding the experiences of frontline public health workers adapting to changing community immunization needs during a global pandemic is crucial to inform workforce capacity and organizational decision-making for the next mass immunization campaign. Prior studies focus on short-term or simulated experiences [10, 17–22], and systems-level analyses from a human factors perspective [16], providing unique insights into workflow processes to support clinic staff. Previous qualitative work has also richly explored the experiences of mass vaccination clinic volunteers, outlining the important factors for supporting role satisfaction [23]. There is a need to supplement this knowledge with the sustained experiences of mass vaccination clinic staff working through an unpredictable global pandemic such as the one caused by SARS-CoV-2. Therefore, the purpose of this study is to explore the evolving experiences of a multi-disciplinary sample of participants working in the COVID-19 mass vaccination clinics in the Region of Waterloo between July 2021 and April 2022.

## Methods

### Study design, selection of participants and data collection technique

This qualitative study explored the dynamics of COVID-19 mass vaccination clinics. The participants were purposively recruited clinic staff that worked at the mass vaccination clinics in the Region of Waterloo [24, 25]. They worked at several sites/clinic types, including three large clinics, one large pop-up clinic, one small clinic and various mobile pop-up clinics throughout the community. An initial recruitment email was sent to all clinic staff soliciting their interest in sharing their experiences at the clinics. Initially, of those who expressed interest in the study, those with the earliest availability to be interviewed across the different clinics (two from each clinic) were first interviewed. This ensured clinic staff diversity in the selection process. Subsequently, while still paying attention to ensuring representativeness across the clinic types, staff who expressed interest were interviewed until saturation was reached in the data [26, 27]. Saturation was agreed upon through weekly research team debriefs that involved the lead researchers (MT and RT), who led the data collection process, sharing their insights from the completed interviews. The Region of Waterloo is located in southwestern Ontario, Canada, and has a population of 620 000 residents as of 2020, with a mix of urban and rural communities [28].

A total of 16 volunteers were individually interviewed in English for 45–60 min virtually via Microsoft Teams. Each interview was audio recorded and automatically transcribed verbatim in preparation for analysis. Simultaneously, MA and AB supported cleaning the transcripts, removing any direct identifiers and correcting the automated version while listening to the recording, if needed. After each transcript was cleaned, they were exported to Taguette 1.3.0 for qualitative analysis to aid the coding process. After conducting the first three interviews, MT and RT reflected on their interview notes and discussed emerging issues that needed further exploration. These discussions not only helped to identify points of saturation [26] but also helped in further refining the interview guide and probes.

### Data analysis

MT led the data analysis process. He worked collaboratively with RT, MA and AB. The process started with each of the four researchers separately coding two transcripts, which informed the development of a codebook that was agreed upon by holding discussions to establish points of convergence and departure. The discussions involved renaming, removing or merging some codes to arrive at the joint codebook. The open coding was then continued by MA and AB by applying the generated codebook to the remaining transcripts. If new codes were generated, these were jointly shared by email, and an agreement to include them (or not) in the codebook was reached at weekly review data analysis meetings.

The final codebook contained 230 open codes; MT, RT, MA and AB reviewed the final codes and each separately selected codes that they deemed relevant to exploring the adaptive dynamics of the COVID-19 mass vaccination clinic context. A discussion to review the selected codes was organized with all authors; this yielded a selection of 100 codes that were jointly agreed upon as relevant to the study subject. The 100 codes were then grouped and regrouped by MT, RT, MA and AB through an iterative process that included weekly reviews and discussions to agree on the groupings. This yielded themes and sub-themes applicable to the subject of analysis. Next, the completed, thematically organized codes were shared with all the authors for review and sense-making. This process involved discussions with senior team members (NW, CMB and KG) and led to an agreement on the results, which all authors reviewed to provide varied and rich insights.

## Results

**Fig. 1:**
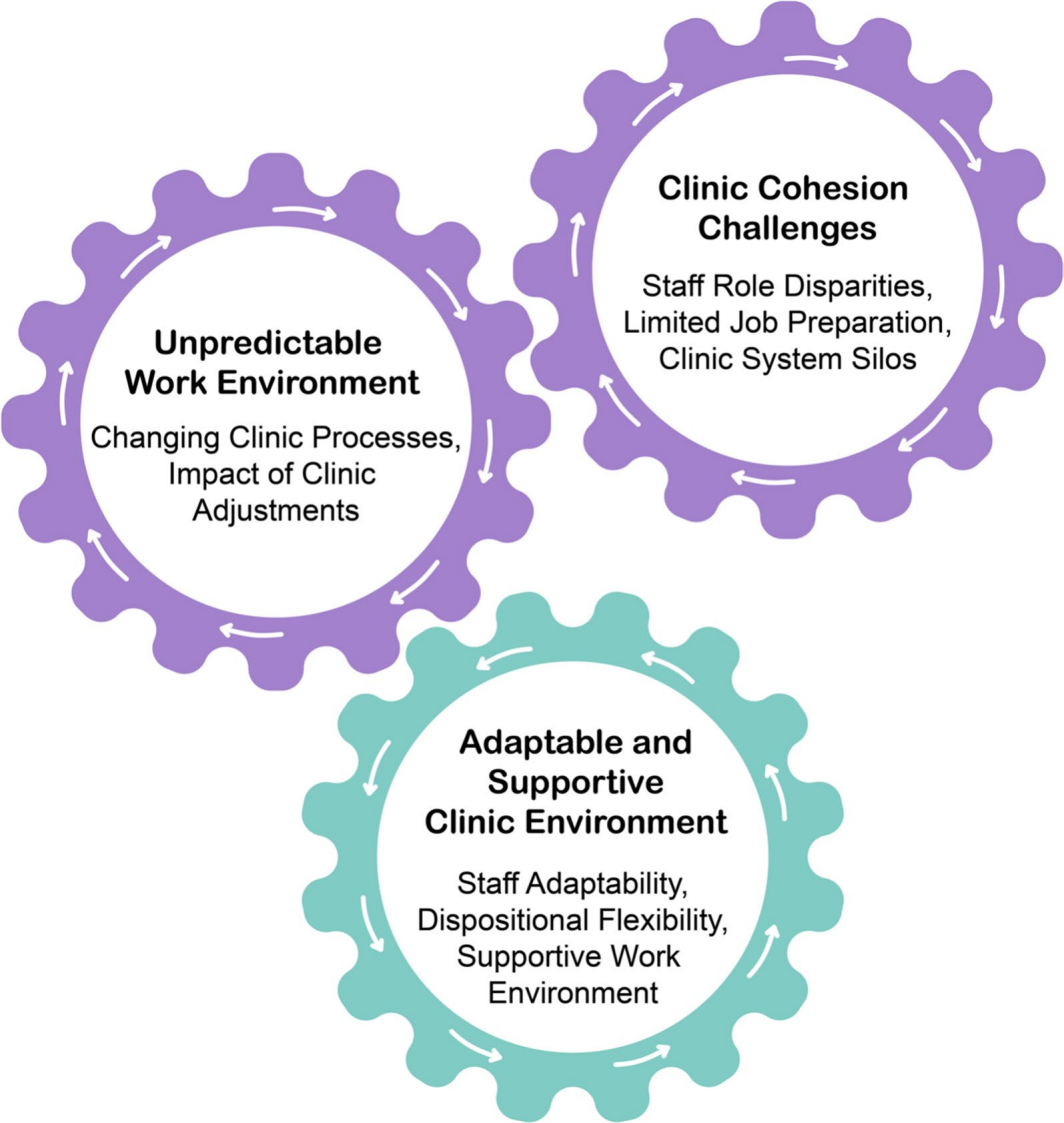
Interrelated factors affecting the dynamics of COVID-19 mass vaccination clinics

Three interrelated themes were developed from the qualitative analysis (Fig. 1). While the clinic staff struggled to establish successful clinics amid an unpredictable work environment and challenges with clinic cohesion, they created adaptable and supportive clinic environments to operate COVID-19 mass vaccination clinics successfully. What follows are insights into the difficulties in balancing the need for agile emergency responses with informed and supported staff.

### Unpredictable work environment

A common analogy used by participants was “flying a plane and building [it] at the same time” (Interviewee 008). While noting that the “plane did fly”, this analogy highlights the unpredictable work environment theme that clinic staff navigated within the clinics. This theme is explained by two subthemes: changing clinic processes and the impact of clinic adjustments. Unplanned changes to staff schedules and clinic processes impacted clinic staff, contributing to an unpredictable work environment. As shown in Fig. 1, this theme and the limitations to the clinic cohesion theme hindered the optimal implementation of the COVID-19 mass vaccination clinics in the Region of Waterloo.

#### Changing clinic processes

Changes to clinic processes, such as vaccine eligibility, screening procedures, vaccine delivery techniques and staff roles, contributed to unpredictable changes to clinic workflow and processes. Many of these modifications were due to the rapidly changing nature of the pandemic. While this was recognized by clinic staff, many noted that the experience felt “like a rollercoaster”.

“The clinic was going through a bit of a transformation. Honestly, it was always transforming.” (Interviewee 001).

“We have to go with the newest things every time, like things are changing all the time.” (Interviewee 003).

Participants found their roles dynamic and variable, resulting from evolving vaccine clinic modalities, including mass vaccination clinics, pop-up clinics, drive-thru clinics and vaccination buses. They transitioned between roles throughout the day based on the current state of the pandemic and the specific needs of the clinic environment. Participants had to be prepared to cover multiple positions when needed, contributing to an unpredictable work environment. For example, supervisors noted that, on some days, they would start the day welcoming clients to the facility and later find themselves covering at the clinic check-out station.

“There is no consistency. There is still no actual job description for the roles since the pandemic is evolving, and everything has evolved with it.” (Interviewee 007).

#### Impact of clinic adjustments

Towards the beginning of the mass immunization campaign, participants explained that the clinics were often overstaffed, which created an unnecessarily hectic atmosphere. Some participants felt they were only needed for part of their shift and suggested scheduling staff for shorter shifts.

“The place was literally crawling with staff. It was overstaffed like crazy for the first little while.” (Interviewee 008).

As the immunization campaign evolved, clinic supervisors expressed concerns about the stress and burnout of clinic staff and the potential impact this could have on the safety and effectiveness of the clinic. They had busy schedules and working long hours, often without breaks or weekends off, to maximize community immunizations.

“I was burning out. After working 13–15 h every day for weeks on end without a weekend off, I didn’t want a clinic environment where you had management staff who ended up calling in sick… who weren’t taking breaks because they had too much going on.” (Interviewee 002).

Generally, an ongoing contention was that these adjustments made it challenging to know what to expect and plan for each day in the clinic.

“At times, the response was reactionary, and that often led to a domino effect of other unforeseen consequences.” (Interviewee 006).

### Clinic cohesion challenges

Clinic cohesion challenges included three sub-themes: staff role disparities, limited job preparation and clinic system silos.

#### Staff role disparities

The clinic staff included health professionals with many backgrounds, such as nurses, pharmacists, family doctors, retired surgeons and paramedics. Given the diverse professional backgrounds of the clinic staff, one source of indirect conflict was differences in compensation. Participants noted pay discrepancies between physicians and other immunizers, suggesting that physician efforts should be redirected towards other areas of the pandemic response.

“We found that doctors were being paid [much more] to do the same job as a nurse. As a nurse, I was not happy to hear that… it’s a waste of healthcare finances. They should be taking care of patients and relieving the load of the nurses in clinics or hospitals.” (Interviewee 001).

“During business hours, doctors are paid $170 or $220 an hour to vaccinate… as a citizen, as a taxpayer, as someone who values the healthcare system at large, I think that that was foolish and unfair.” (Interviewee 002).

Further, conflicts between clinic staff and supervisors were another limiting factor in clinic cohesion. Disagreements often arose about using scarce resources, such as opening a vaccine vial towards the end of the clinic’s scheduled hours. In addition, running a clinic when vaccine supplies were low was considered an unnecessary use of resources.

“If we had challenges with vaccination supply, then we shouldn’t have run a clinic. We should have complemented the staffing [at another clinic]. We ended up using resources that were unnecessary.” (Interviewee 003).

Participants reflected on the mobile clinics – some deployed on retrofitted public transit buses parked in an accessible location – that operated differently than the mass vaccination clinics, and how this impacted staff capacity and burnout. While mass vaccination clinic cohesion was disrupted when staff were overburdened, mobile clinics, with smaller staff numbers, were more easily overwhelmed and stressed.

“[For the first mobile clinics], we had a small team of nurses that were not COVID deployed yet. They were overburdened and over capacity. They exceeded the actual hours they were supposed to be working on a weekly basis.” (Interviewee 006).

#### Limited job preparation

In our study, clinic staff participants reflected on their journey within the evolving mass vaccination clinics. “Hitting the ground running” was a common sentiment among participants and may have contributed to the staff role disparities described above:

“You were flying a plane and building it at the same time.” (Interviewee 008).

“We were pretty much everything. We were a nurse. We were also [human resources]. We were also [information technology support]. We were security.” (Interviewee 003).

Planning the procedures and operations of the mobile clinics during their implementation was challenging for clinic supervisors, particularly when onboarding other staff. The participants with this experience initially felt they had limited knowledge about operating a mobile clinic, leading to feeling overwhelmed and anxious, in addition to pressures from public health to get doses into arms.

“At face value, it looked like it was a well-oiled machine. I’d say it was probably some of the highest anxiety I’ve ever been through.” (Interviewee 003).

Due to the novelty of the COVID-19 mass vaccination clinics, role preparation was challenging. Towards the beginning of the mass vaccination effort, participants experienced the chaotic nature of the clinic environment with little formal training and preparation opportunities.

“It was chaos because it was the start of the pandemic… We all did our best. The readings helped, but doing it in practice was where I got most of my real training done.” (Interviewee 008).

Participants mentioned public health authorities provided virtual training materials. They also said there was no checklist of training documents, videos and activities to review. In addition, most of the training occurred on the job, with participants drawing on their previous clinical and administrative experiences to succeed in their roles. Clinic supervisor participants felt they had insufficient training resources and preparation to lead the mass vaccination clinics.

“There was no training at all. None. If I were to give feedback to public health, [I would say] there was a significant gap as there were no resources provided to clinic supervisors or managers.” (Interviewee 003).

In addition, scope definition presented a significant challenge with clinic staff contributing their skills in different roles as needed versus in a planned manner.

“I think the biggest challenge or the thing that we didn’t do well was that scope definition. I think everybody stepped up and did what needed to be done to get it done.” (Interviewee 006).

#### Clinic system silos

When examining the participants’ clinic experiences from a systems perspective, clinic supervisors expressed that the mass vaccination clinics operated in silos, despite similar objectives.

“We’re very siloed. We ran our clinic separately from the [other clinics]. It wasn’t very cohesive.” (Interviewee 003).

The participants revealed limited communication between clinic supervisors, managers and Public Health authorities, especially in the first few months of clinic operations. The lack of clear direction was particularly prominent in the mobile clinics where the timing of planning and implementation coincided:

“With the mobile clinics, pretty much all of it beyond the actual immunizing was unclear. It was being planned while it was being implemented, so there was not a lot of experienced staff behind it.” (Interviewee 009).

“We had to set up our own emergency response [for the mobile clinic]. We had to create our own policy code because that was not provided. We had to order our own epinephrine and blood pressure cuff. We weren’t even provided with that.” (Interviewee 003).

### Adaptable and supportive clinic environment

The staff coped with the unpredictable and challenging environment through adaptability, dispositional flexibility and fostering a supportive work environment.

### Staff adaptability

The clinic staff willingly adapted to changing circumstances as the pandemic progressed. Clinic staff participants reflected on learning new information about the vaccines and clinic processes, which enabled them to operate efficiently in a coordinated manner even when circumstances changed.

“We definitely learned as we went, but the circumstances have changed so much.” (Interviewee 009).

Over time, clinic staff became familiar with clinic processes and found it easier to adapt to changes. For example, these changes were often communicated through team huddles, allowing all clinic staff to align with changing roles and expectations.

“We have a quick huddle… Just a heads up for things that shift whether it was needle changes or just interval changes or population expectations for that day, etc.” (Interviewee 004).

Further, adaptability regarding staff schedules was another critical component in the success of the mass vaccine clinics. Considering changing clinic circumstances, clinic staff participants emphasized the importance of taking time to recharge. Clinic staff were also allowed to change their schedules as needed, contributing to an accommodating and supportive work environment.

“Knowing that I can change my schedule is nice… I don’t have to worry about letting them know that’s going to happen.” (Interviewee 001).

#### Dispositional flexibility

Dispositional flexibility referred to how the clinic staff simultaneously acknowledged challenging situations yet saw these as an opportunity to improve. The pandemic created a unique environment where typical public health clinic flow would not apply. Participants reflected on the thought they put into clinic flow design to accommodate changing demographics of eligible clients, for example, ensuring that children, who were only eligible for the Pfizer-BioNTech Comirnaty COVID-19 vaccine, did not mistakenly receive a dose of the Moderna SpikeVax COVID-19 vaccine.

“We had to put in a fail-safe so that no one could get Moderna instead of Pfizer when they were 14 years old… We would only have certain rows who could do the 11 to 18’s.” (Interviewee 007).

This flexibility was also demonstrated in the participants’ views of the dynamic nature of the COVID-19 mass vaccination clinics. Over time, they viewed the changes as beneficial and less disruptive rather than contributing to chaotic situations. Additionally, a common notion of continuous learning, “figuring it out as you go”, was strongly emphasized.

“I wouldn’t want to be on an airplane that’s being built while it’s taking off for flight. I think we did better than that. I think that our plane was built. We were just tuning it as we were flying.” (Interviewee 002).

“There was some training… but since the job didn’t exist before, there was a lot of learning on the go and developing the role itself as everything progressed.” (Interviewee 007).

Contingency planning was essential to adapt to unexpected changes in clinic operations, such as technical failures and extreme weather events. For instance, clients would line up inside the clinic building instead of on the pavement in the case of rain or snow. Further, paper processes and technical support were contingencies put in place should the clinic experience issues with the coronavirus disease of 2019 vaccines global access – Ontario (COVaxON), the COVID-19 vaccination management system in Ontario.

“We have good workarounds in place. Whenever [COVaxON] went down… we had contingencies built in like paper processes and lots of super user support on the floor to help staff.” (Interviewee 004).

Similarly, as clinic operations progressed, clinic supervisors could create functional teams with well-defined roles and scope. They achieved this by developing policies and procedures for process issues, documentation and training.

“We have a really well-oiled system now, right? Like without having an official checklist. We have this team now that has defined scope, and that was missing for most of this rollout.” (Interviewee 006).

#### Supportive work environment

All clinic staff participants reported a positive experience working in the COVID-19 mass vaccination clinics, owing to a supportive work environment. Contributing factors to a positive experience included supportive colleagues, management and effective communication. Ultimately, this helped all clinic staff participants feel that their work was meaningful and valued by the people around them.

“Overall, I would say that it’s been a positive experience working at the clinics. The staff have been very nice people to work with. Management is friendly. They do their best to communicate with us.” (Interviewee 009).

“Our clinic ran very well. We had a very strong team. We selected the right staff to help move people through.” (Interviewee 003).

Clinic staff valued having friendly supervisors who instilled confidence in their teams, achieved through clear communication about roles and responsibilities, as well as openness to providing support and accepting feedback. Effective communication between clinic supervisors and other staff strengthened team dynamics. Transparency and open communication with staff through huddles were greatly appreciated by clinic staff.

“It’s honestly worked out really well here. Like everyone knows their responsibilities. My manager is amazing…” (Interviewee 005).

“We had this meeting to get ourselves organized and stuff… It made a big difference in terms of morale. And we have a lot of support.” (Interviewee 003).

Within the broader context of the pandemic, all clinic staff felt that their work was meaningful. Although working in the clinics was challenging and, at times, stressful, staff participants felt motivated and described their role as the “exit strategy for the pandemic” (*Interviewee* 008).

“I often described the clinic as part of the exit strategy for the pandemic. And I think that feeling was the motivation I needed.” (Interviewee 008).

## Discussion

In this study, we explored clinic staff participants’ perspectives on the dynamics of vaccination clinics during the evolving COVID-19 pandemic. The unpredictable work environment described the instability experienced by mass vaccination clinic staff. The challenges of clinic cohesion generated similar counterproductive circumstances under which the clinic staff worked. Nonetheless, individual and system-level adaptations led participants to observe the mass vaccination clinics as a largely successful, fulfilling and rewarding experience.

Considering the challenges described in this paper, we draw some key lessons. First, to cope with a crisis, establishing a routine is a crucial strategy. The quality of the work environment is a known predictor of the likelihood of teams achieving a common goal. The literature has highlighted how a stable routine with established roles and responsibilities enables building basic skill sets while simultaneously creating opportunities for building cohesion within teams [29, 30]. Participants working at mass vaccination clinics in this study found it difficult to quickly establish routine and stability owing to the unpredictable nature of the COVID-19 pandemic that continues to result in continuously changing vaccination protocols [31]. As a result, clinic staff roles also evolved, leaving staff unprepared and learning in a busy environment. This feeling created stress points and sometimes led to suboptimal performance, similar to experiences reported in Canada during the swine flu (H1N1) pandemic in 2009 [14]. Therefore, while routine is undoubtedly essential, clinic staff should be prepared for crises requiring additional flexibility while ensuring “routine” periods of recovery and stability are also included.

A second lesson learned is that trying to ensure clinics always have enough personnel can result in a few staff members being stretched too thin across various shifts. This can cause them to feel overworked and lead to staff redundancy and burnout, which can, in turn, negatively impact their motivation and productivity [32–34]. At the start of the immunization campaign, the uncertainty around supply and demand sometimes led clinics to be overstaffed, creating inefficiency. Later, as the vaccine supply increased, staff were scheduled for more shifts, which led to feelings of overwork [35]. Certainty and flexible work schedules boost staff motivation and increase productivity [36, 37]. During periods of low demand, it is recommended to utilize time effectively by providing staff with preparation or refresher training for the various roles they may need to fulfil during the vaccination period, as well as participating in team-building activities [38, 39]. In other studies [40, 41], during pandemic periods, staff are often assigned emergent roles for which they need more preparation, as also noted in this study. Creating clinics with inclusive and adaptive planning can play a crucial role in building favourable work environments and optimizing resource utilization, particularly during a pandemic [42].

A third lesson learned is the importance of immunization campaign planners and leadership actively participating in the clinics during the design and development of operational procedures. As highlighted in other studies, engaged leaders are better equipped to collect and respond to feedback from clinic staff and navigate top–down and bottom–up planning approaches [43, 44]. Having leadership embedded in the clinics can help identify the challenges highlighted in this study, including role disparities among staff, inadequate job preparation and the isolation of clinic systems. By being present and involved, leaders can take appropriate measures to address these issues and ensure that the clinics run smoothly and efficiently for the staff and the clients.

Fourth, to address staffing disparities that can limit clinic staff team cohesion, it is essential to have clear and well-defined staff roles, eliminate pay inequities and ensure workload-to-staff balance. Clarity of staff roles is essential in streamlining organizational operations, increasing staff role satisfaction and fostering team cohesiveness [45]. In addition, fair payment of staff is a known motivation factor that plays a significant role in ensuring sustained motivation of mass vaccination clinic staff [46, 47]. This requires paying attention to differences in pay for the same work to avoid pay inequities. Further, adequate staffing levels that match the workload available are also important, as this can help reduce staff resentment and ensure that everyone can manage their workload effectively. By addressing these staffing disparities, clinics can create a more positive and productive work environment for all staff members.

Fifth, research has shown that adequately preparing clinic staff can bring about significant benefits [35, 36, 48–50]. However, during a pandemic, limited job preparation can be expected due to the rapid deployment of an immunization campaign. Therefore, it is recommended that training should be anticipated as “learning on the job” as much as possible. Learning by doing is a well-established capacity-building approach [50, 51], and even in a crisis, active learning should be deliberately designed to include specific phases of reflection and education to achieve its intended outcomes better. It is important to note that, when clinic staff feel less competent, it affects their confidence, leading to increased stress, anxiety and reduced performance. Conversely, if clinic staff feel confident in their skills and abilities, their well-being and work outputs improve [52–55]. Thus, it is essential to prioritize comprehensive training programs that allow staff to feel secure and competent, especially during pandemic situations. This can be achieved through online and in-person training and observation sessions, regular feedback and opportunities for staff to suggest recommendations and support from supervisors and colleagues. By providing adequate support and training, clinic staff can perform their jobs effectively, efficiently and with the utmost care for clients and each other.

Sixth, one significant challenge faced by clinics is the limitation of cross-training due to the silos that exist within the system of clinics of a particular geographical region. This can create difficulties in providing support as nuanced clinic operations and procedures must be learned, leading to a lack of flexibility and adaptability in unexpected situations such as pandemics. To address this, it is essential to create a well-coordinated system that enables learning across clinics. Peer-to-peer learning has been shown to be highly effective in increasing resource efficiency and effectiveness [56, 57], and as such, it is important to consider leveraging this approach to strengthen the capabilities of clinic staff. By fostering a culture of learning and collaboration within mass vaccination clinics within a region or area, individual clinics can better prepare and support each other in the face of future challenges such as delays in vaccine delivery, increased community demand, supply chain disruptions or changes in required pandemic safety measures, thereby consistently delivering the best possible outcomes for clients.

Lastly, during times of pandemics, it is important for workplaces to be adaptable. This is because the environment can become unpredictable, and team cohesion may be challenged [58]. In this study, giving staff more control over their schedules eased frustrations and contributed to more team cohesion. This environment allowed for team-based problem-solving, improving operational processes and vaccination capacity. Therefore, it is important for clinic staff to be prepared to handle unpredictable environments and have the necessary skills to be resilient [48, 59, 60]. Increased resilience displayed by staff may contribute to increased public confidence in response teams during emergencies. Additionally, having a supportive and friendly work environment during a crisis can benefit staff well-being and lead to a sense of meaningfulness in the work they are doing, ultimately contributing to greater productivity. By being flexible and open to new ideas and ways of working as vaccination guidelines change, mass vaccination clinics can continue to provide their essential service while supporting staff and client safety and well-being, even in the face of an ongoing pandemic.

## Methodological considerations

The trustworthiness of our study is based on four factors: credibility, transferability, dependability and confirmability [61]. To achieve credibility, our study interviewees were purposively selected across all mass immunization clinics in the Region of Waterloo. Care was taken to ensure that the staff selected had worked at the clinics for most of the time they had been in operation by the time of the interviews. This enabled us to capture more grounded experiences of working at the clinics. We have also provided a detailed account of our study setting and methods of inquiry and analysis, ensuring the transferability of our study findings or at least the methods of inquiry. The dependability of our study findings is anchored through all authors actively participating in a collaborative process of study design, data collection, analysis and manuscript writing. To attain conformability, our study tools and findings were shared with Public Health officials from the Region of Waterloo, who took an active role in the setup and running of the mass immunization clinics. In addition, some of the authors (KG, NW and RT) worked at some of the clinics. This not only validated the findings but ensured the accurate presentation of the local context.

## Conclusion and recommendations

Effectively managing the COVID-19 pandemic has required public health officials to coordinate “flying and building a plane simultaneously” – a seemingly impossible task. Nevertheless, immunization clinics have provided vaccines to entire communities, which is a great achievement. Valuable feedback from the clinic staff participants in this study suggests that better predicting clinic operations and staff schedules, or acknowledging that there is room for improvement and open-mindedness towards feedback, would help to improve the functioning of mass vaccination clinics. Similarly, reducing staff role disparities is key to increasing staff satisfaction and productivity. Creating this through an inclusive, forward-thinking yet adaptive planning mechanism can create more positive experiences for clinic staff. To improve team cohesion, role definitions and preparation should be planned, and opportunities of low demand should be used to increase competencies among staff for different roles. Team cohesion can also be supported through more inclusive work schedules and by encouraging participation in team-building activities. Harnessing diversity by promoting peer–peer learning can increase resource efficiency and effectiveness. An adaptable and supportive clinic environment was crucial in countering the challenges of running dynamic mass COVID-19 clinics in the Region of Waterloo. Preparing clinic staff with essential skills for adaptability is critical, and we recommend that it is included as part of hiring criteria and on-the-job training.

As the world faces the ongoing challenges posed by the COVID-19 pandemic, it is crucial that we draw on the lessons learned from pandemic work experiences, including COVID-19 and past pandemics where similar lessons were learned, such as the swine flu (H1N1) pandemic in 2009 [14]. The findings of this study highlight the critical importance of leveraging real-world experiences to better prepare for future emergencies and avoid repeating past mistakes. By sharing evidence and collaborating with public health authorities and policy-makers, we can strengthen our crisis management strategies and improve our response to similar crises in the future. It is essential that we take a proactive approach to pandemic management to minimize the impact of such emergencies and protect the health and well-being of our communities.

## Data Availability

Transcripts of the interview are available upon request to the corresponding author.

## Abbreviations

COVID-19: Coronavirus disease 2019
SARS-Cov-2: Severe acute respiratory syndrome coronavirus 2
H1N1: Swine flu
